# Supplementary material for: Content-rich biological network constructed by mining PubMed abstracts
Source: BMC Bioinformatics. 2004 Oct 8;5:147. doi: 10.1186/1471-2105-5-147 (PMC528731; doi:10.1186/1471-2105-5-147)
Supplement: Additional File 2 — The original results of the above study (non-essential files are deleted to keep the file size under the limit set by BMC bioinformatics). [file 1471-2105-5-147-S2.bz2 › chilibotAdditionalFile2/dip10/212ID7768894E718/html/CSNK2B_CSNK2A2.html]

 


 **CSNK2B** and **CSNK2A2** 
  
Found 141 abstracts in PubMed, retrieved 10.  
 

 What does Google say? 
 PDF only 
| .edu only 

---

**Interactive relationship** (e.g. stimulation, inhibition, etc)

**Neutral relationship**- We found that the stress induced kinase casein kinase CK II  [ **CSNK2A2** ]   [ **CSNK2B** ]  phosphorylates the Ser276 immediately preceding the tyrosine motif, increasing AQP4 mu 3A interaction and enhancing AQP4 lysosomal targeting and degradation.  Ref: 11742978 EMBO J, 2001

**Non-interactive relationship** (e.g. studied together, co-existance, homology, etc.)

- Rhodnius CK II  [ **CSNK2A2** ]   [ **CSNK2B** ]  is strongly inhibited by heparin Ki = 9 nM and uses ATP Km = 36 microM or GTP Km = 86 microM as phosphate donors.  Ref: 12110292 Insect Biochem Mol Biol, 2002
- In addition, the phosphorylation of the recombinant N in vitro can be blocked by a CK II  [ **CSNK2A2** ]   [ **CSNK2B** ]  inhibitor, heparin.  Ref: 12711319 Biochem Biophys Res Commun, 2003
- hondai on the activities of four distinct Ser Thr protein kinases two casein kinases CK I and CK II  [ **CSNK2A2** ]   [ **CSNK2B** ] , A kinase and C kinase were determined in vitro.  Ref: 12612451 Biol Pharm Bull, 2003
- These results, therefore, indicate that the purified kinase is a CK II  [ **CSNK2A2** ]   [ **CSNK2B** ]  protein kinase and may be involved in the regulation of DNA topoisomerase I activity.  Ref: 12243354 Mol Cells, 2002
- CONCLUSION Our results suggest that CK II  [ **CSNK2A2** ]   [ **CSNK2B** ]  plays an important role in cellular proliferation and the mechanism of its activation.  Ref: 12017291 Anticancer Res< MedlineTA>Anticancer Res, 2002
- In this study we show that Vitellin VT phosphorylation in chorionated oocytes of Rhodnius prolixus is completely inhibited by heparin 10 microg ml, a classical casein kinase II CK II  [ **CSNK2A2** ]   [ **CSNK2B** ]  inhibitor.  Ref: 12110292 Insect Biochem Mol Biol, 2002
- The recombinant N was phosphorylated by BHK cellular extracts and by purified CK II  [ **CSNK2A2** ]   [ **CSNK2B** ] .  Ref: 12711319 Biochem Biophys Res Commun, 2003
- Thus, our data demonstrate that cellular CK II  [ **CSNK2A2** ]   [ **CSNK2B** ]  phosphorylates rabies virus N.  Ref: 12711319 Biochem Biophys Res Commun, 2003
- The activity range of CK II  [ **CSNK2A2** ]   [ **CSNK2B** ]  was between 500 1500 U mg protein.  Ref: 12017291 Anticancer Res< MedlineTA>Anticancer Res, 2003
- One unit of CK II  [ **CSNK2A2** ]   [ **CSNK2B** ]  activity is defined as the amount catalysing the transfer of 1 pmol 32P into the substrate within 1 minute at 37 degrees C.  Ref: 12017291 Anticancer Res< MedlineTA>Anticancer Res, 2003
- The reading frame encodes a polypeptide consisting of 526 amino acid residues, containing a number of DNA binding motifs and phosphorylation sites for PKC, CK II  [ **CSNK2A2** ]   [ **CSNK2B** ] , and p34cdc2.  Ref: 12736779 J Mol Med< MedlineTA>J Mol Med, 2003
- We have obtained a 3000 fold VT free enriched preparation of CK II  [ **CSNK2A2** ]   [ **CSNK2B** ] .  Ref: 12110292 Insect Biochem Mol Biol, 2002
- Characterization of a CK II  [ **CSNK2A2** ]   [ **CSNK2B** ]  protein kinase from etiolated oat seedlings.  Ref: 12243354 Mol Cells, 2002
- Incubation of VT with purified Rhodnius CK II  [ **CSNK2A2** ]   [ **CSNK2B** ]  and 32 P ATP led to the incorporation of 2 mols of phosphate mol VT.  Ref: 12110292 Insect Biochem Mol Biol, 2002
- Enhanced casein kinase II CK II  [ **CSNK2A2** ]   [ **CSNK2B** ]  activity in human lung tumours.  Ref: 12017291 Anticancer Res< MedlineTA>Anticancer Res, 2002
- Physical, biochemical and antigenic properties of this kinase identify it as casein kinase II CK II  [ **CSNK2A2** ]   [ **CSNK2B** ] .  Ref: 11861906 Nucleic Acids Res, 2002
- BACKGROUND Casein kinase II CK II  [ **CSNK2A2** ]   [ **CSNK2B** ]  is expressed at a higher level in lung tumours when compared to the corresponding non neoplastic lung tissue.  Ref: 12017291 Anticancer Res< MedlineTA>Anticancer Res, 2002
- Furthermore, N phosphorylation in the virus infected cells can be inhibited by a CK II  [ **CSNK2A2** ]   [ **CSNK2B** ]  specific inhibitor, dichloro beta D ribofuranosyl benzimidazole.  Ref: 12711319 Biochem Biophys Res Commun, 2003
- The respective serines are located in conserved recognition sites for luminal protein kinases protein kinase CK II  [ **CSNK2A2** ]   [ **CSNK2B** ]  and Golgi casein kinase.  Ref: 12033762 Virology, 2002
- MATERIALS AND METHODS The aim of the present study was to evaluate the activity of CK II  [ **CSNK2A2** ]   [ **CSNK2B** ]  both in neoplastic and non neoplastic human lung tissue by using a synthetic peptide with Arg Arg Arg Asp Asp Asp Ser Asp Asp Asp RRRDDDSDDD sequence.  Ref: 12017291 Anticancer Res< MedlineTA>Anticancer Res, 2002
- The monoclonal antibodies CK II  [ **CSNK2A2** ]   [ **CSNK2B** ]  and A45 B B3 were used with the immuno cytochemical standard method for detecting IDT BM.  Ref: 12678519 Int J Surg Investig, 2000
- The same mutations also abolish phosphorylation of eIF5 when transfected into mammalian cells suggesting that CK II  [ **CSNK2A2** ]   [ **CSNK2B** ]  phosphorylates eIF5 at these two serine residues in vivo as well.  Ref: 11861906 Nucleic Acids Res, 2002
- These data show that an insect yolk protein contain phosphorylation sites for a cyclic nucleotide independent protein kinase such as CK II  [ **CSNK2A2** ]   [ **CSNK2B** ] .  Ref: 12110292 Insect Biochem Mol Biol, 2002
